# Theoretical Analysis of the *n*_1_ν_1_ + ν_3_ Combination Bands in Hydrogen Bihalide Anions, XHX^–^, X = {F, Cl, Br, and I}

**DOI:** 10.1021/acs.jpca.5c05801

**Published:** 2025-11-14

**Authors:** Jake A. Tan, Jer-Lai Kuo

**Affiliations:** † Department of Chemistry, 6491 University of West Florida, Pensacola, Florida 32514, United States; ‡ Institute of Atomic and Molecular Science, 38017 Academia Sinica, Taipei 10617, Taiwan

## Abstract

The anharmonic vibrational structures of XHX^–^ anions, X = {F, Cl, Br, and I}, were examined with a focus on the *n*_1_ν_1_ + ν_3_ combination bands, where *n*_1_ is the excitation quanta of the symmetric X–H stretch (ν_1_) and ν_3_ is the asymmetric X–H stretch. Full-dimensional potential energy surfaces (PESs) were built by using a multilevel scheme based on CCSD­(T) and MP2 levels of *ab initio* theories. These dual-level PESs were then used to simulate the anharmonic spectra and to assess the extent of vibrational coupling. The vibrational signatures of XHX^–^ are very similar to those of the previously studied proton-bound noble gas dimers. The combination bands become more visible as the halide becomes heavier. The quantum nature of these combination bands was examined using an adiabatic model. Furthermore, the halide binding energies for XHX^–^, X = {F, Cl, Br, and I}, were studied using a modern fragmentation-based method, energy decomposition analysis based on absolutely localized molecular orbitals (ALMO-EDA). The halide binding energy decreases from that of FHF^–^ to that of IHI^–^. Both the geometric distortion and dispersion-free frozen components destabilize these complexes, weakening their binding energies. However, the dispersion, polarization effects, and charge transfer contributions stabilize these complexes.

## Graphical abstract

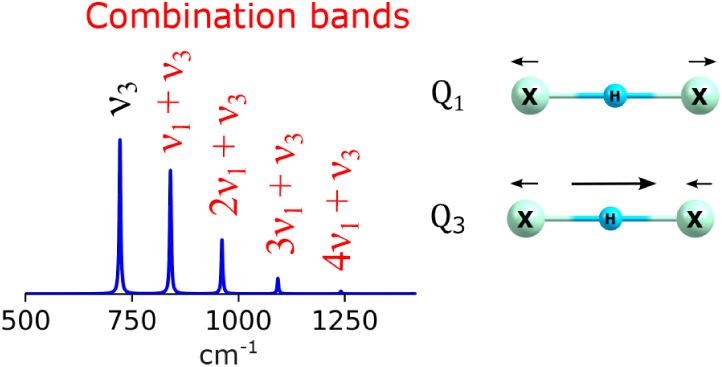

## 1 Introduction

The hydrogen bihalide anions, XHX^–^, X = {F, Cl, Br, and I}, represent a prototypical complex in which two anions surround a proton. These species are of general interest, as they represent the simplest structures for ionic hydrogen bonding. Compared with neutral hydrogen bonds, ionic hydrogen bonds are much stronger. Their strengths are reported to be in the 5–35 kcal/mol range.^1–3^

Among the XHX^–^, the FHF^–^ ion has received significant attention. Several studies have been conducted with regard to its structure, bonding, and spectra.^4–48^ Recently, Dereka and coworkers^4^ reported a thorough joint experimental and theoretical study on FHF^–^. Using two-dimensional infrared spectroscopy (2D IR) and VSCF/VCI calculations, they concluded that the bonding in FHF^–^ is represented as a “crossover from hydrogen to chemical bonding.″ Dunning^5^ utilized spin-coupled generalized valence bond (SCGVB) theory, which is an advanced orbital theory, to reinvestigate the bonding motif in FHF^–^. The results have indicated that the bonding in FHF^–^ cannot be attributed to a traditional hydrogen bond nor to a traditional covalent bond. Instead, the results have suggested a new bonding motif in which the two polarized fluoride anions are held by the H^+^.

The infrared spectra of XHX^–^ have been measured and reported in the literature.^4,29–49^ These spectra are usually measured in noble gas matrices,^29–44^ but a few gas-phase measurements^46–48^ have also been reported for FHF^–^, ClHCl^–^, and BrHBr^–^. Ault^30,31^ measured the FHF^–^ infrared spectrum in an Ar matrix. Based on their spectrum, the asymmetric H–F stretch (ν_3_) appears as a strong band at 1364 cm^–1^, while the degenerate H^+^ bend appears as a moderate band at 1217 cm^–1^. Meanwhile, Hunt and Andrews^32^ also measured the spectrum of FHF^–^ in the Ar matrix and assigned the 1377 cm^–1^ band as ν_3_, while the band at 1908 cm^–1^ was tentatively assigned to the ν_1_ + ν_3_ combination band. They also reported a combination band that involves one quantum of the symmetric and asymmetric H–F stretches (ν_1_ + ν_3_). These peaks agree reasonably well with the gas-phase measurement by Kawaguchi and Hirota,^46^ where the ν_3_ band is at 1331 cm^–1^, while the ν_1_ + ν_3_ band is at 1848 cm^–1^. It is likely that the difference in peak position for the ν_3_ band between these studies can be attributed to the effect of the matrix.

As for the ClHCl^–^ anion, there are also several reported matrix isolation studies.^34–39,45,47^ The peak positions of the ν_3_ band in the Ne, Ar, Kr, and Xe matrices have all been reported. It is interesting to note that the ν_3_ band is sensitive to the matrix environment. In particular, the ν_3_ band is located at 729 cm^–1^ (Ne matrix),^38^ 696 cm^–1^ (Ar matrix),^34–37,39,45^ 663 cm^–1^ (Kr matrix),^37,39,45^ and 644 cm^–1^ (Xe matrix).^45^ These studies indicate the critical role of the matrix in the spectral peak positions. Among these measurements, the ν_3_ band in the Ne matrix is very close to the gas-phase measurement by Kawaguchi,^47^ where the ν_3_ band is observed at 723 cm^–1^. Similarly, the ν_1_ + ν_3_ peak is also found to red-shift as the matrix is changed from Ne to Xe. The ν_1_ + ν_3_ peak positions are 993 cm^–1^ (Ne matrix),^38^ 955 cm^–1^ (Ar matrix),^35^ 916 cm^–1^ (Kr matrix),^37^ and 893 cm^–1^ (Xe matrix).^37^

The ν_3_ peak in BrHBr^–^ is located at a lower frequency.^37,40–42,48^ Bondybey and coworkers^40^ have measured the infrared signatures of the BrHBr^–^ ion. A mixture of Ar, HBr, and Br_2_ was passed through a glow discharge, and the resulting product was deposited at 20 K. The infrared spectrum of the deposit revealed peaks at 727, 892, and 1053 cm^–1^. Bondybey and coworkers^40^ had initially thought that these peaks are attributed to the BrHBr radical trapped in an Ar matrix. Later Milligan and Jacox^41^ revisited the spectrum and presented convincing evidence that those peaks are attributed to the BrHBr^–^ ion. The band assignments were then updated to 727 cm^–1^ (ν_3_), 892 cm^–1^ (ν_1_ + ν_3_), and 1053 cm^–1^ (2ν_1_ + ν_3_). Räsänen and coworkers,^37^ on the other hand, measured the BrHBr^–^ infrared spectrum in Kr and Xe matrices. The ν_3_ band was observed at 687 cm^–1^ (Kr matrix) and 646 cm^–1^ (Xe matrix). In addition to the ν_3_ band, they also observed the progression of *n*_1_ν_1_ + ν_3_ combination bands in the 790–1160 cm^–1^ window. Meanwhile, Pivonka^48^ and coworkers measured the action spectrum of Ar·BrHBr^–^. The relevant spectral signatures were 733 cm^–1^ (ν_3_), 890 cm^–1^ (ν_1_ + ν_3_), 1048 cm^–1^ (2ν_1_ + ν_3_), and 1202 cm^–1^ (3ν_1_ + ν_3_). Note that the first three peaks are very close to what Milligan and Jacox observed in the Ar matrix.

As for the IHI^–^ anion, its history with regard to matrix isolation experiments is similar to that BrHBr^–^.^37,42–44^ Noble^43^ detected the IHI^–^ anion trapped in solid Ar. However, Noble initially thought that the spectral signatures could be attributed to the IHI^·^ radical. Eventually, Ellison and Ault^44^ have observed spectral signatures similar to those of Noble when HI and several metal iodides were codeposited in an Ar matrix, which suggests that the spectral signatures are indeed attributed to the M^+^(HI_2_)^−^ species. It was found that the interaction of M^+^ would affect the ν_3_ peak position: 603 cm^–1^ (Na^+^(HI_2_)^−^), 673 cm^–1^ (Cs^+^(HI_2_)^−^). Aside from the Ar matrix , the IHI^–^ anion has also been studied in Ne, Kr, and Xe matrices.^37,42^

Based on these experimental measurements, the appearance of the *n*_1_ν_1_ + ν_3_ combination bands indicates that anharmonicity plays a vital role in understanding the vibrational signatures of XHX^–^. In this work, a high-quality full-dimensional potential energy surface (PES) is built for XHX^–^, X = (F, Cl, Br, and I). These PESs were then used to investigate the anharmonic nature of these species. It is hoped that the present results in this work would motivate more gas-phase measurements for these systems. The vibrational coupling is then evaluated and analyzed by using an intuitive and practical basis set for the vibrational Hamiltonian. Lastly, the binding of X^–^ with HX is also investigated.

## 2 Theoretical and Computational Methods

### 2.1 Geometry Optimization and Frequency Calculations

The ground singlet state geometries for the hydrogen bihalide anions, XHX^–^ (X = F, Cl, Br, and I), were optimized at the coupled cluster single–double and perturbative triple excitations (CCSD­(T)) level of theory.^50–52^ The Dunning^53^ correlation-consistent aug-cc-pVQZ basis set was used for H, F, Cl, and Br atoms, while the aug-cc-pVQZ-PP basis set was used for the I atoms. We note that the PP in the latter basis set is based on a small-core pseudopotential (PP), which was developed by Peterson and coworkers^54,55^ and was obtained from the EMSL Basis Set Exchange Web Page.^56–58^ We note that although a larger basis set would provide a better description of the electronic structure, the use of a quadruple-ζ quality basis set is sufficient to describe the salient spectral features and achieves a good balance between computational cost and accuracy.

A frequency calculation was performed for the optimized structures to confirm that they correspond to a minimum on their respective potential energy surface (PES). In this work, geometry optimizations, frequency calculations, and single-point energy calculations were performed using the Gaussian 09 Rev. E01 program.^59^

### 2.2 Halide Ion Binding Energy Calculations

The stability of hydrogen XHX^–^ with respect to HX and X^–^ in the gas phase can be assessed by examining the energetics of the reaction below:

1 $$XHX_{\left(g\right)}^{-}\to X_{\left(g\right)}^{-}+HX_{\left(g\right)}$$

The binding energy (Δ*E*_Binding_) of X^–^ to HX is expressed as

2 $$\Delta E_{Binding}=E_{X_{\left(g\right)}^{-}}+E_{{HX}_{\left(g\right)}}-E_{{XHX}_{\left(g\right)}^{-}}$$

where *E*_i_ corresponds to the total energy (electronic energy plus nuclear repulsion terms) of the chemical species *i*.

To gain further insights into the nature of the interaction between X^–^ and HX, energy-decomposition analysis based on absolutely localized molecular orbitals (ALMO-EDA)^60^ was performed in Q-Chem 5.4,^61^ using the X^–^ and HX fragments.

### 2.3 Potential Energy Surface (PES) and Dipole Moment Function (DMF) Construction

A full-dimensional potential energy surface (PES) and a dipole moment function (DMF) were constructed in normal mode coordinates for each of the XHX^–^ species. We note that although other coordinates such as internal or localized normal coordinates are equally valid means of representing the vibrational degrees of freedom, our choice of using normal mode coordinates has two advantages. First, the normal mode coordinates are an orthogonal set of coordinates. As a result, there would be no kinetic energy coupling in the vibrational Hamiltonian.^62^ Second, all of the vibrational coupling arising from mechanical anharmonicity is concentrated in the full-dimensional PES.

The set of normal mode coordinates is 1) the symmetric X-H stretch (*Q*_1_), 2) the degenerate H bend (*Q*_2*x*_) and (*Q*_2*y*_), and 3) the asymmetric X–H stretch (*Q*_3_). Figure 1 depicts these coordinates. Both PES and DMS were sampled by performing single-point energy calculations on a set of Gauss-Hermite quadrature grids.^63,64^ Thirteen grid points were used to sample along the *Q*_1_ and *Q*_3_ coordinates, while 11 grid points were used to sample along the degenerate (*Q*_2*x*_) and (*Q*_2*y*_) coordinates. As a result, each PES and DMS is comprised of 20,449 grid points.

**Figure 1:**
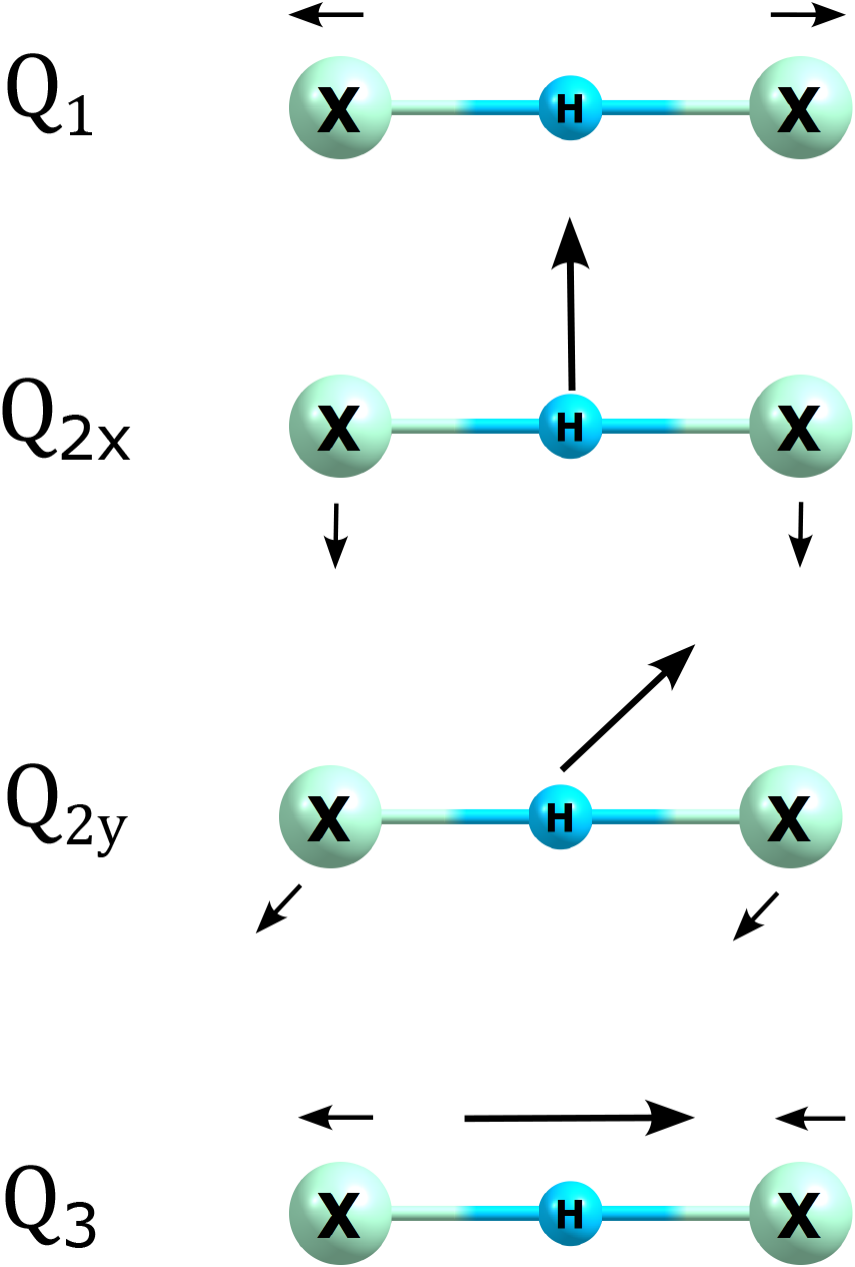
Normal mode coordinates for the XHX^–^ anions.

Performing all 20,449 single-point calculations at the CCSD­(T) using a quadruple-ζ basis set would be expensive. As a result, the PES was built using the n-mode representation (n-MR)^65^ approach coupled with a mixed-level scheme.^66–72^ The spirit of the n-MR approach is similar to that of the many-body expansion. The PES is expressed as the sum of intrinsic k-mode potentials, V̅_k_.

3 $$\mathrm{V̂}\left(\mathrm{Q⃗}\right)={\mathrm{V̅}}_{1}+{\mathrm{V̅}}_{2}+{\mathrm{V̅}}_{3}+{\mathrm{V̅}}_{4}$$

where Q⃗ = (Q_1_, Q_2x_, Q_2y_, Q_3_). V̅_1_ represents a potential energy that accounts for the one-mode anharmonicity and does not contain any intermode coupling. Meanwhile, V̅_2_, V̅_3_, and V̅_4_ would account for the intrinsic two-mode, three-mode, and four-mode couplings, respectively.

An important benefit of using the n-MR approach is that it is possible to reduce the computational cost without compromising the accuracy and quality of the PES. This can be achieved by mixing different levels of theory in building a PES. In this work, the more important V̅_1_ and V̅_2_ terms are built at the CCSD­(T) level of theory, while the less important V̅_3_ and V̅_4_ terms are built using the more affordable MP2 level of theory.

4 $$\mathrm{V̂}\left(\mathrm{Q⃗}\right)={\mathrm{V̅}}_{1}^{CCSD(T)}+{\mathrm{V̅}}_{2}^{CCSD(T)}+{\mathrm{V̅}}_{3}^{MP2}+{\mathrm{V̅}}_{4}^{MP2}$$

Since the DMF is only needed to predict transition intensities and would not affect the peak positions of a calculated spectrum, the DMFs in this work were constructed at the MP2 level of theory.

### 2.4 Anharmonic Vibrational Calculations

The details for the anharmonic vibrational calculations have been described elsewhere.^73–77^ As a result, only the salient features of the method will be recapitulated in this work. The vibrational Hamiltonian used for the anharmonic calculations takes the form

5 $$Ĥ=\frac{-\hbar^{2}}{2}\overset{k}{\underset{i=1}{\sum }}\frac{1}{\mu_{i}}\frac{\partial^{2}}{\partial Q_{i}^{2}}+\mathrm{V̂}\left(\mathrm{Q⃗}\right)$$

We note that the above Hamiltonian is a simplification of the Watson Hamiltonian.^78^ The vibrational angular momentum terms are neglected in the present treatment. We believed the effect of vibrational angular momentum would not change the salient features of the simulated spectrum, which are the ν_3_ and the *n*_1_ν_1_ + ν_3_ combination bands. Also, most of the available measurements for the hydrogen bihalides were conducted through matrix isolation experiments and are not rotationally resolved. However, we are aware that the vibrational angular momentum can lead to *l*-type doubling, which would then split the doubly degenerate bending modes.

We note that since the normal mode coordinates used in this work are expressed in terms of Cartesian displacements and not mass-weighted Cartesian displacements, a nonunit reduced mass (μ_i_) for each vibrational degree of freedom *Q*_i_ is introduced.^73,79^ From here, the vibrational Schrödinger equation was solved by using a discrete variable representation (DVR) in the harmonic oscillator basis.^64,80–84^ The peak positions are then obtained by referencing the eigenvalues with respect to the ground state. To predict the corresponding intensity at each transition, the integrated absorption coefficient^85^ (*A*_f0_) was calculated using the formula below:

6 $$A_{f0}=\left(\frac{\pi N_{A}}{3c\varepsilon_{0}\hbar^{2}}\right)\nu_{f0}{|\langle \psi_{0}|\mathrm{\mu ̂}|\psi_{f}\rangle |}^{2}$$

where *N*_A_ is Avogadro’s constant, *c* is the speed of light in vacuum, ε_0_ is the permittivity in vacuum, *ℏ* is the reduced Planck’s constant, and ν_f0_ is the transition wavenumber between the initial ψ_0_ and final ψ_f_ states. The integral ⟨ ψ_0_ | μ̂ | ψ_f_ ⟩ is the transition dipole moment.

## 3 Results and Discussion

### 3.1 Structure of the Hydrogen Bihalide (XHX^–^) Anions

The optimized structure for all XHX^–^ anions is linear and centrosymmetric (*D*_∞h_ point group). The proton (H^+^) is sandwiched between two halide (X^–^) anions. Such a structure can be easily rationalized if one thinks of the XHX^–^ formation from X^–^ and HX. In the HX molecule, the bond is polar such that the H atom possesses a partially positive charge, while the X atom possesses a partially negative charge. As a result, the X^–^ anion would interact favorably with the H atom.

Table 1 shows the structural parameters for the XHX^–^ anions. The X–H distance (*R*_X‑H_) increases from FHF^–^ to IHI^–^. Such a trend is easily understood based on the radius of the X^–^ ion. From F^–^ to I^–^, the halide’s radius increases. Hence, it is anticipated that *R*_X‑H_ would increase down the halogen family.

**Table 1:** CCSD­(T) Structural Parameters and Harmonic Frequencies for XHX^–^

Species	*R*_X‑H_ (Å)	θ(^◦^)	ν_1_ (cm^–1^)	ν_2_ (cm^–1^)	ν_3_ (cm^–1^)
FHF^–^ ^a^	1.1394	180.0	639	1347	1199
ClHCl^–^ ^a^	1.5598	180.0	342	834	413
BrHBr^–^ ^a^	1.6976	180.0	210	643	679
IHI^–^ ^b^	1.8897	180.0	147	694	684

a Aug-cc-pVQZ basis set was used for H, F, Cl, and Br atoms.

b Aug-cc-pVQZ-PP basis set was used for I atoms.

In order to assess the quality of the present CCSD­(T) calculations in predicting structural parameters, a comparison to the measured gas-phase structural parameters is made. Only FHF^–^ and ClHCl^–^ have available experimental structural parameters in the gas phase. Based on Kawaguchi’s gas-phase studies,^46,47^ the equilibrium F–F distance is 2.2777 Å, while the equilibrium Cl–Cl distance is 3.1122(26) Å. The present calculations agree well with those values. In particular, the calculated X–X bond distances are 2.2788 Å for F–F and 3.1196 Å for Cl–Cl.

### 3.2 The Nature of Interaction between X^–^ and HX in X···H···X^–^

Table 2 shows the raw binding energy (Δ*E*_Binding_) needed to dissociate the XHX^–^ anions into X^–^ and HX. These raw binding energies are calculated by considering only the total energy of each species. The raw Δ*E*_Binding_ values in kcal/mol are as follows: FHF^–^ (44.57), ClHCl^–^ (23.56), BrHBr^–^ (22.53), and IHI^–^ (19.13). Due to the use of a finite basis set, the binding energy calculations suffer from basis set superposition error (BSSE).^86^ As a result, these raw binding energies are overestimated. Counterpoise^87^ (CP) calculations were performed in order to assess the extent of BSSE. We found that the BSSE increases as the halide gets bulkier. The calculated BSSE in kcal/mol is as follows: FHF^–^ (0.95), ClHCl^–^ (0.79), BrHBr^–^ (3.26), and IHI^–^ (4.42). Since BSSE^86^ tends to overestimate the binding energies, the effect of CP correction is to decrease these binding energies as shown in Table 2.

**Table 2:** CCSD­(T) Halide Binding Energies in kcal/mol for XHX^–^ ^a^ ^b^

	Δ*E*_Binding_ (kcal/mol)			
Process	Raw	CP Correction	CP and Harmonic Corrections	CP and Anharmonic ZPE Corrections
${\mathrm{FHF}}_{\left(g\right)}^{-}\to F_{\left(g\right)}^{-}+{\mathrm{HF}}_{\left(g\right)}$	44.57	43.62	43.06	42.81
${\mathrm{ClHCl}}_{\left(g\right)}^{-}\to {\mathrm{Cl}}_{\left(g\right)}^{-}+{\mathrm{HCl}}_{\left(g\right)}$	23.56	22.77	23.58	23.14
${\mathrm{BrHBr}}_{\left(g\right)}^{-}\to {\mathrm{Br}}_{\left(g\right)}^{-}+{\mathrm{HBr}}_{\left(g\right)}$	22.53	19.27	19.71	19.52
${\mathrm{IHI}}_{\left(g\right)}^{-}\to I_{\left(g\right)}^{-}+{\mathrm{HI}}_{\left(g\right)}$	19.13	14.71	14.95	14.88

a Aug-cc-pVQZ basis set was used for H, F, Cl, and Br atoms.

b Aug-cc-pVQZ-PP basis set was used for I atoms.

The effect of the vibrational zero-point energy (ZPE) on the binding energies is examined at two levels. The first level involves ZPE correction at the harmonic level, while the second level involves ZPE correction at the anharmonic level. As shown in Table 2, the harmonic ZPE shifts the CP-corrected Δ*E*_Binding_ by 0.81 kcal/mol at most. To explore the effect of anharmonicity, the potential energy curves for the XH species were sampled with 13 grid points along the normal coordinate, and the anharmonic ZPE was calculated by using the DVR method. The resulting counterpoise and anharmonic ZPE-corrected (CP + anharm ZPE) Δ*E*_Binding_ are also shown in Table 2. Consideration of the anharmonic effects would slightly lower the Δ*E*_Binding_ by 0.44 kcal/mol at most with respect to the CP and harmonic-corrected (CP + harmonic corrected) Δ*E*_Binding_. We note that between BSSE and ZPE, the former has a greater effect on Δ*E*_Binding_.

These calculated binding energies are good indicators of the XHX^–^ hydrogen bond strength. It is evident in Table 2 that across all scenarios, the Δ*E*_Binding_ decreases from FHF^–^ to IHI^–^. Such a trend is consistent with Larson and McMahon’s^16^ measurement of the X^–^ binding enthalpies with HX using ion cyclotron resonance halide-exchange measurements. The binding enthalpies are FHF^–^ (38.6 kcal/mol), ClHCl^–^ (23.1 kcal/mol), and BrHBr^–^ (20.0 kcal/mol). As for the IHI^–^ ion, Caldwell and Kebarle^2^ determined the binding enthalpy to be 17.0 kcal/mol. The trends for the binding energy and enthalpy for XHX^–^ can be rationalized using Pearson’s hard and soft acids and bases (HSAB) principle.^88,89^ The proton (H^+^) is a hard acid due to its small size and high charge density. Hence, H^+^ prefers to interact with hard bases. Among the halides (X^–^), the degree of hardness decreases in the order of F^–^ > Cl^–^ > Br^–^ > I^–^. As a result, the X···H···X^–^ interaction energy is expected to be strongest in FHF^–^ and would decrease down the halogen group.

Since the XHX^–^ anions are hydrogen-bonded systems, it is worthwhile to study the interaction between the X^–^ and H···X moieties of the XHX^–^ ion through fragmentation-based methods. As a result, we performed ALMO-EDA^60,90^ calculations using the CCSD­(T) geometries at the ωB97M-V level of theory. The aug-cc-pVQZ basis set was used for the H, F, Cl, and Br atoms, while the aug-cc-pVQZ-PP basis set was used for the I atoms. In the ALMO-EDA method,^60^ the binding energy (Δ*E*_Binding_) is expressed as

7 $$\Delta E_{Binding}=\Delta E_{GD}+\Delta E_{DFFRZ}+\Delta E_{DISP}+\Delta E_{POL}+\Delta E_{CT}$$

where Δ*E*_GD_ is the geometric distortion (GD) energy, which is the change in energy as the fragment changes its geometry from the isolated monomer to the complex. Δ*E*_DFFRZ_ is the dispersion-free frozen energy (DFFRZ). It contains contributions from electrostatic interactions and Pauli repulsion. Meanwhile, Δ*E*_DISP_ is the dispersion (DISP) effect, Δ*E*_POL_ is the polarization (POL) effect, and Δ*E*_CT_ is the charge transfer (CT) contribution. We note that a negative ALMO-EDA component indicates stabilization, while a positive component indicates destabilization.

Figure 2 shows the components of ALMO-EDA for the ωB97M-V binding energies. Across all the XHX^–^ anions, the GD (red bars) and DFFRZ (purple bars) destabilize the hydrogen bihalides. From FHF^–^ to IHI^–^, the GD contribution decreases, while the DFFRZ contribution increases. The DISP (yellow bars), POL (blue bars), and CT (green bars) tend to stabilize the hydrogen bihalide. The stabilization is dominated by the POL and CT contributions. It is interesting to note that the average contribution of POL and CT to the stabilization is roughly constant across the species. However, the contribution of the DFFRZ to the destabilization increases from FHF^–^ to IHI^–^. As a result, we conclude that the DFFRZ destabilizes these complexes and is the main reason why the binding energy decreases with heavier halide ions, as shown by the black bars in Figure 2. Using the classical frozen decomposition^91^ formalism, the DFFRZ can be separated into electrostatic interaction (ELEC) and Pauli repulsion (PAULI). Decomposing the DFFRZ reveals that although the electrostatic interaction stabilizes the XHX^–^, such stabilization is negated by a greater Pauli repulsion. The values for the ALMO-EDA are available in Table S1.

**Figure 2:**
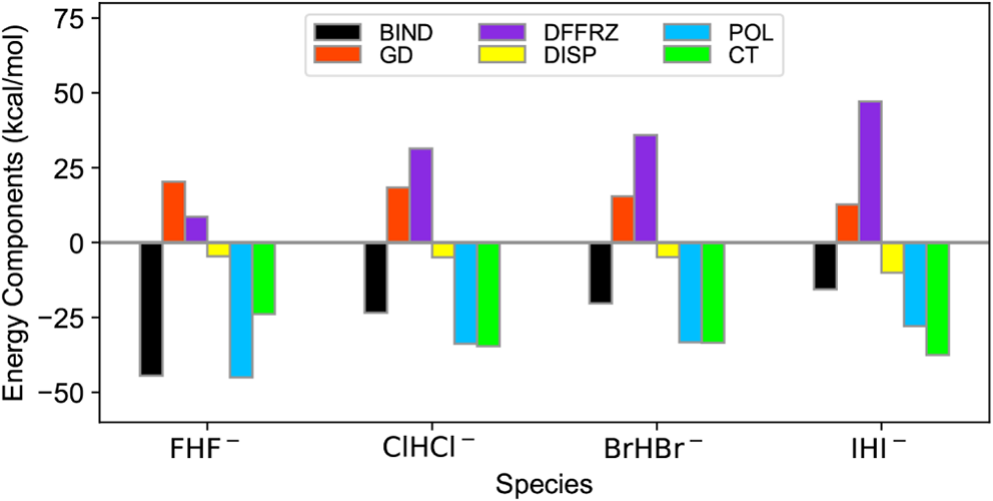
ALMO-EDA for the XHX^–^ anions.

We now examine how the electron density is distributed among the atoms in the XHX^–^ ion through Mulliken population analysis at the ωB97M-V level of theory. Table S2 shows the Mulliken net atomic charges for H and X atoms in an XHX^–^ ion. The atomic charge for the H atom (*Q*_H_) becomes more negative from FHF^–^ to IHI^–^. In particular, the values for *Q*_H_ are 0.575 (FHF^–^), 0.056 (ClHCl^–^), −0.149 (BrHBr^–^), and −0.250 (IHI^–^). Meanwhile, the charge of the X atoms (*Q*_X_) becomes more positive (less negative) from FHF^–^ to IHI^–^. The values for *Q*_X_ are −0.788 (FHF^–^), −0.528 (ClHCl^–^), −0.425 (BrHBr^–^), and −0.375 (IHI^–^). These trends in *Q*_H_ and *Q*_X_ suggest that the extent of charge transfer increases as the halide gets heavier. As a result, the charge difference in an H–X bond decreases from FHF^–^ to IHI^–^, which indicates that the covalent character of the X–H bond increases from FHF^–^ to IHI^–^.

Lastly, we present a brief discussion of the bonding motif in XHX^–^. Using qualitative molecular orbital (MO) theory and DFT calculations, Hoffmann and coworkers^92^ showed that the bonding in XHX^–^ can be understood by examining the orbital interaction between the H^+^ and the neighboring X^–^ ions. The 1*s* orbital of H^+^ can interact with the group orbitals formed from the linear combination of the *p*_*z*_ orbitals of X^–^. From these three atomic orbitals, three molecular orbitals are formed. These are the sigma bonding (σ_b_), sigma nonbonding (σ_nb_), and sigma antibonding $\left(\sigma_{ab}^{*}\right)$ . Both σ_b_ and σ_nb_ are doubly occupied, while the $\sigma_{ab}^{*}$ is unoccupied. As a result, the bonding motif in XHX^–^ can be regarded as a three-center, four-electron (3c-4e) bond. Figure S1 shows the qualitative MO diagram.

### 3.3 Vibrational Spectrum for XHX^–^ (X = F, Cl, Br, and I)

As mentioned above, the XHX^–^ ion has four vibrational degrees of freedom. Table 3 shows the labels used in this work, together with their corresponding symmetry labels. Their corresponding fundamental bands are labeled as follows: symmetric X–H stretch (ν_1_), degenerate H bend (ν_2_), and asymmetric X–H stretch (ν_3_). The ν_1_ band belongs to the $\Sigma_{g}^{+}$ representation. As a result, it is a dark band (no intensity) in the infrared region. The present anharmonic calculations predicted that the ν_1_ bands for XHX^–^ are as follows: FHF^–^ (585 cm^–1^), ClHCl^–^ (304 cm^–1^), BrHBr^–^ (191 cm^–1^), and IHI^–^ (135 cm^–1^). This decreasing trend for the ν_1_ band from FHF^–^ to IHI^–^ can be rationalized in terms of the mass of the halide. As the halide becomes heavier, the reduced mass (μ_1_) increases, which then lowers the vibrational frequency for the ν_1_ band.

**Table 3:** Mode Labels for XHX^–^

Label	Symmetry Species	Description
ν_1_	$\Sigma_{g}^{+}$	Symmetric X–H stretch
ν_2_	Π_u_	H bend
ν_3_	$\Sigma_{u}^{+}$	Asymmetric X–H stretch

Meanwhile, the ν_2_ band belongs to the doubly degenerate Π_u_ representation and is expected to be a bright band. The present anharmonic calculations that predicted the positions for the ν_2_ bands are as follows: FHF^–^ (1297 cm^–1^), ClHCl^–^ (801 cm^–1^), BrHBr^–^ (716 cm^–1^), and IHI^–^ (631 cm^–1^). We note that this decreasing frequency trend is an indication that the strength of hydrogen bonding decreases from FHF^–^ to IHI^–^ and is consistent with the halide binding energy in Table 2. Furthermore, the ν_2_ intensity was also found to decrease from that of FHF^–^ to that of IHI^–^. The calculated ν_2_ anharmonic intensities are FHF^–^ (125 km/mol), ClHCl^–^ (11 km/mol), BrHBr^–^ (2 km/mol), and IHI^–^ (0 km/mol). As shown in Figure 3, only the ν_2_ band for FHF^–^ has an appreciable intensity. This indicates that the change in the dipole moment along the bend coordinate decreases as the halide gets heavier. We note that similar behavior has been observed in the case of proton-bound noble gas dimers.^73^

**Figure 3:**
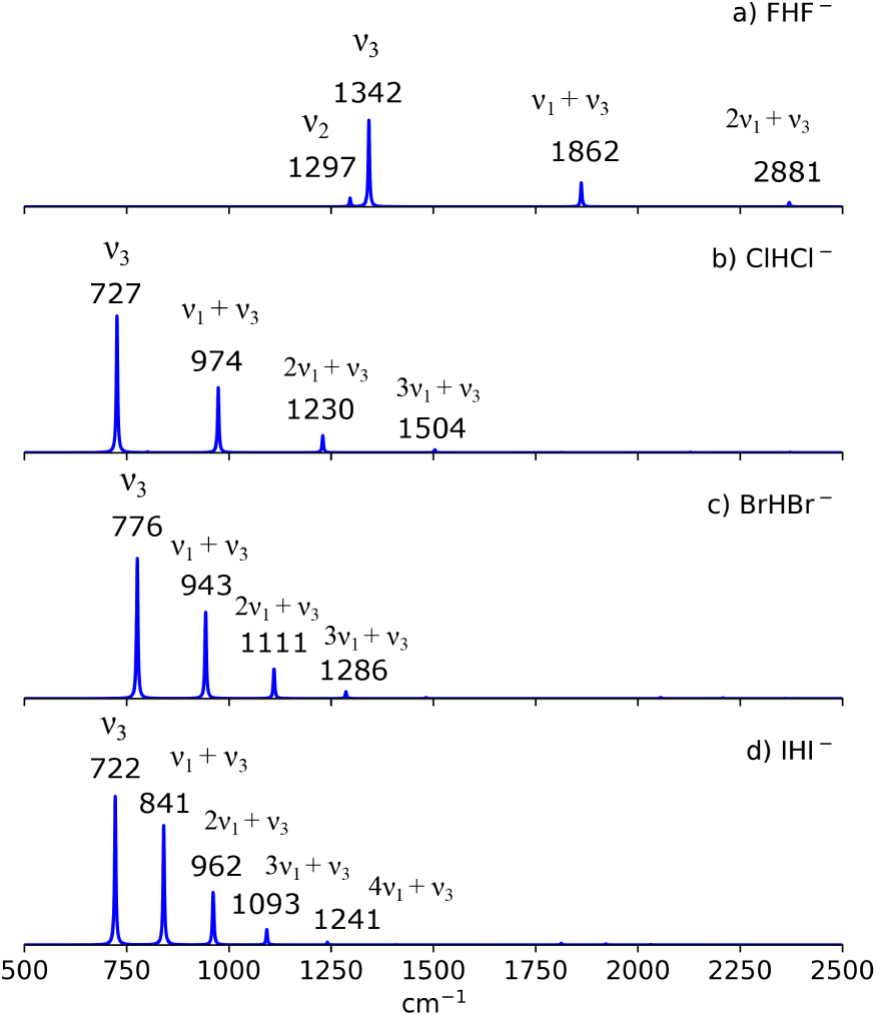
Anharmonic spectra for the XHX^–^ anions.

The ν_3_ band belongs to the $\Sigma_{u}^{+}$ representation. As this band corresponds to the asymmetric X–H stretch, the change in the dipole moment is large along this motion. As a result, the ν_3_ band possesses the greatest band intensity as depicted in Figure 3. The calculated anharmonic frequencies for the ν_3_ band are as follows: FHF^–^ (1342 cm^–1^), ClHCl^–^ (727 cm^–1^), BrHBr^–^ (776 cm^–1^), and IHI^–^ (722 cm^–1^). These ν_3_ anharmonic frequencies are all blueshifted when compared to their harmonic counterparts in Table 1.

In addition to the intense ν_3_ band, Figure 3 also shows additional bright bands to the right of the ν_3_ band. These bands are equally spaced, and their spacing is comparable to their respective ν_1_ frequency. As a result, these additional bands correspond to the *n*_1_ν_1_ + ν_3_ combination bands, where *n*_1_ represents the excitation quantum for ν_1_. Furthermore, since these combination bands also belong to the $\Sigma_{u}^{+}$ representation, their corresponding wave functions can mix with that of the ν_3_ band, which leads to intensity redistribution in the spectrum. Kawaguchi and Hirota^46^ verified that this is indeed the case when they performed a diode laser spectroscopy experiment for FHF^–^. Based on their findings, ν_3_ is located at 1331 cm^–1^, while ν_1_ + ν_3_ is located at 1846 cm^–1^. The simulation presented here agrees with these experimental bands within 31 cm^–1^. As shown in Figure 3a, the ν_3_ band is predicted at 1342 cm^–1^, while the ν_1_ + ν_3_ band is predicted at 1862 cm^–1^. Meanwhile, 2ν_1_ + ν_3_ is predicted at 2370 cm^–1^ with a weaker intensity.

The anharmonicity in FHF^–^ has been studied by several groups.^8–14,22,27^ An earlier study by Lohr and Sloboda^8^ indicated that higher-order terms (quartic and biquadratic) are important in describing the proton vibration in FHF^–^. Spirko and coworkers^10^ performed two-dimensional simulations for FHF^–^ and reported that the anharmonic vibrational frequencies are very sensitive with respect to the method used to treat electron correlation. Del Bene and coworkers^27^ did a two-dimensional (2D) simulation using a PES comprised of 247 points at the CCSD­(T)/aug’-cc-pVTZ level of theory but predicted the ν_3_ band at a higher frequency (1476 cm^–1^). Botschwina^22^ utilized the CCSD­(T*)-F12b approach to build a high-quality three-dimensional (3D) PES and simulate the vibrational states of FHF^–^. We note that our calculated peak positions for ν_3_ and ν_1_ + ν_3_ agree within 12 cm^–1^ when compared with Botschwina’s results.

We now examine the ν_3_ and *n*_1_ν_1_ + ν_3_ bands in ClHCl^–^ (Figure 3b). From FHF^–^ to ClHCl^–^, the reduced mass along the *Q*_1_ coordinate increases, which then lowers the ν_1_ stretch frequency. As a result, the spacing among the progression bands gets narrower, and a more efficient intensity redistribution is seen. The strongest band in the ClHCl^–^ spectrum is the ν_3_ band, which is located at 727 cm^–1^. To the right of this band are the ν_1_ + ν_3_ (974 cm^–1^) and 2ν_1_ + ν_3_ (1230 cm^–1^) combination bands. The 3ν_1_ + ν_3_ band has a very weak intensity at 1504 cm^–1^. Comparing these anharmonic frequencies with the earlier experiment by Kawaguchi^47^ shows that the present simulation agrees well with the measured peak position for the ν_3_ band at 723 cm^–1^. Forney and coworkers^38^ measured the spectrum of ClHCl^–^ in the Ne matrix. The ν_3_ band is at 728.9 cm^–1^, and the ν_1_ + ν_3_ band is at 992.6 cm^–1^. These peak positions are in reasonable agreement with the present work, and part of the discrepancy can be attributed to the effect of the matrix. In the Kr and Xe matrices, the ν_3_ band can go down below 665 cm^–1^.^37^

Simulations^15,19,20,27^ on the vibrational spectrum of ClHCl^–^ has also been discussed in the literature. Ikuta and coworkers^20^ performed a one-dimensional and two-dimensional anharmonic calculations that involve the symmetric and antisymmetric H–Cl stretch using the MP4SDTQ/DZ+(d,p) level of theory and basis set. They found that the asymmetric H–Cl stretch is highly anharmonic. The 1D anharmonic frequency is 283 cm^–1^ higher than the harmonic frequency. Furthermore, they found that there is strong potential coupling between the symmetric and asymmetric H–Cl stretch. A 2D simulation would lower the ν_3_ peak by 121 cm^–1^. Such a finding was also verified in our previous work^73,93–96^ on proton-bound dimers. Meanwhile, Del Bene and Jordan^27^ performed a similar 2D calculation with a PES comprised of 270 points at CCSD­(T)/aug’-cc-pVTZ. Their predicted frequencies for the ν_3_ and ν_1_ + ν_3_ bands are 776 cm^–1^ and 1043 cm^–1^, respectively. Anharmonic rovibrational calculations were also performed by Sebald and coworkers^15^ based on a CCSD­(T*)-F12b PES built using a quintuple-ζ quality basis set. The ν_3_ frequency is predicted at 723 cm^–1^, while the ν_1_ + ν_3_ frequency is 983 cm^–1^.

We now focus on the BrHBr^–^ anharmonic spectrum (Figure 3c). The anharmonic ν_1_ band for BrHBr^–^ is almost 33% lower than that of the ClHCl^–^ anion. Therefore, the spacing among the progression bands becomes even narrower in BrHBr^–^. This in effect would further enhance the intensity distribution among the progression bands. As shown in Figure 3c, the progression bands have an appreciable intensity up to the 3ν_1_ + ν_1_ band. For BrHBr^–^, the present anharmonic calculation predicted the following bands: ν_3_ (776 cm^–1^), ν_1_ + ν_3_ (943 cm^–1^), 2ν_1_ + ν_3_ (1111 cm^–1^), and 3ν_1_ + ν_3_ (1286 cm^–1^). These results, when compared with the action spectrum of Ar·BrHBr^–^,^48^ show that the predicted bands are blueshifted. The ν_3_ band in the action spectrum is 733 cm^–1^, while the present anharmonic ν_3_ band frequency is 776 cm^–1^. Meanwhile, the discrepancy between the simulation and action spectra for the *n*_1_ν_1_ + ν_3_ combination bands can be as large as 84 cm^–1^. In order to address this discrepancy, it might be worthwhile to simulate the vibrational spectrum of the Ar·BrHBr^–^ ion. We note that although the infrared spectrum of the BrHBr^–^ ion has been measured in noble gas matrices,^37,40–42^ the matrix effects could have shifted the peak positions between 645 and 753 cm^–1^.

Lastly, among the XHX^–^ anions considered in this work, the IHI^–^ anion has the lowest ν_1_ frequency (135 cm^–1^). Such a small ν_1_ frequency would provide an excellent energy matching between the ν_3_ fundamental band and the ν_1_ + ν_3_ combination band, which leads to even more efficient mixing of the wave functions. In fact, the resulting wave function for the ν_1_ + ν_3_ combination band contains significant ν_3_ wave function character such that the ν_1_ + ν_3_ band’s intensity is about 80% of that of the ν_3_ band’s intensity (Figure 3d). The peak positions of the bands are ν_3_ (722 cm^–1^), ν_1_ + ν_3_ (841 cm^–1^), 2ν_1_ + ν_3_ (962 cm^–1^), and 3ν_1_ + ν_3_ (1093 cm^–1^). The anharmonic intensities for the XHX^–^ can be found in Supporting Information.

Unfortunately, the gas-phase spectrum for IHI^–^ has not yet been measured in the literature. We note that the present results for the ν_3_ band are 50 cm^–1^ higher when compared with the measurement in the Ne matrix.^42^ We speculate that the discrepancy could have come from two possible sources. First, this study neglected the vibrational angular momentum terms in the Hamiltonian. The effect of rotational–vibrational interaction can affect the peak positions for IHI^–^. Second, the use of a pseudopotential (PP) for the iodine atom might introduce errors in the PES and the calculated anharmonic frequencies. To see how the use of a PP can affect the vibrational frequencies, we recalculated the anaharmonic spectrum for BrHBr^–^ using aug-cc-pVQZ-PP for Br. A comparison of the anharmonic spectrum is available in Figure S2. We found that using a PP in BrHBr^–^ can blueshift the ν_3_ band by 45 cm^–1^.

In order to understand the quantum nature of these couplings, the matrix representation of the four-dimensional Hamiltonian in eq 5 was expressed in the direct product basis |*n*_1_, *n*_2_, *n*_3_⟩ from the lower-dimensional Hamiltonians. The |*n*_1_, *n*_2_, *n*_3_⟩ basis set is defined as

8 $$\left|n_{1},n_{2},n_{3}\right\rangle \equiv \left|n_{1}\right\rangle ⊗\left|n_{2}\right\rangle ⊗\left|n_{3}\right\rangle$$

where |*n*_1_⟩ is the one-dimensional wave function along the *Q*_1_ coordinate, |*n*_2_⟩ is the two-dimensional wave function along the doubly degenerate *Q*_2*x*_ and *Q*_2*y*_ coordinates, and |*n*_3_⟩ is the one-dimensional wave function along the *Q*_3_ coordinate. From a group theory perspective, both the |0, 0, 1⟩ and |*n*_1_, 0, 1⟩ states belong to the $\Sigma_{u}^{+}$ representation. Hence, their corresponding matrix elements, ⟨*n*_1_, 0, 1|Ĥ|0, 0, 1⟩, are not zero.

Table 4 shows the matrix elements, ⟨*n*_1_, 0, 1|Ĥ|0, 0, 1⟩. From these matrix elements, the quantum nature of these progression bands can be understood. A common feature among these vibrational Hamiltonian matrices is that every |*n*_1_, 0, 1⟩ state couples strongly with its adjacent |*n*_1_ ± 1, 0, 1⟩ states. As a result, the bright |0, 0, 1⟩ state is mixed with the |*n*_1_, 0, 1⟩ states, which makes the transition intensities for the combination bands appreciably intense.

**Table 4:** Vibrational Hamiltonian Matrix Elements for XHX^–^ in the |*n*_1_, *n*_2_, *n*_3_⟩ Basis

FHF^–^	|0, 0, 1⟩	|1, 0, 1⟩	|2, 0, 1⟩	|3, 0, 1⟩
⟨0, 0, 1|	1677			
⟨1, 0, 1|	–377	2307		
⟨2, 0, 1|	–60	528	2933	
⟨3, 0, 1|	10	–102	–641	3556

ClHCl^–^	|0, 0, 1⟩	|1, 0, 1⟩	|2, 0, 1⟩	|3, 0, 1⟩
⟨0, 0, 1|	979			
⟨1, 0, 1|	–222	1318		
⟨2, 0, 1|	–28	313	1657	
⟨3, 0, 1|	4	–48	–380	1995

BrHBr^–^	|0, 0, 1⟩	|1, 0, 1⟩	|2, 0, 1⟩	|3, 0, 1⟩
⟨0, 0, 1|	988			
⟨1, 0, 1|	–169	1196		
⟨2, 0, 1|	–16	238	1403	
⟨3, 0, 1|	2	–27	–291	1610

IHI^–^	|0, 0, 1⟩	|1, 0, 1⟩	|2, 0, 1⟩	|3, 0, 1⟩
⟨0, 0, 1|	927			
⟨1, 0, 1|	–142	1073		
⟨2, 0, 1|	–13	200	1220	
⟨3, 0, 1|	–1	22	244	1366

We also note that as the halide gets bulkier, the magnitude of the off-diagonal elements in the vibrational Hamiltonian matrix ⟨*n*_1_, 0, 1|Ĥ|0, 0, 1⟩ decreases. Although this trend alone would suggest that the vibrational coupling should decrease from FHF^–^ to IHI^–^, an opposite trend in the coupling was observed. The vibrational coupling increases from FHF^–^ to IHI^–^. Such an outcome is understood if one examines the spacing or energy gap between adjacent diagonal elements in Table 4. The spacing decreases from FHF^–^ to IHI^–^, which effectively compensates for the decrease in off-diagonal elements. We note that this pattern for the matrix elements of the vibrational Hamiltonian is similar to that of the proton-bound noble gas dimers.^73,97^

Lastly, the anharmonic spectrum in Figure 3 shows an anomaly in the trend in ν_3_ from FHF^–^ to IHI^–^. The ν_3_ band redshifts from FHF^–^ (1342 cm^–1^) to ClHCl^–^ (727 cm^–1^) but blueshifts from ClHCl^–^ (727 cm^–1^) to BrHBr^–^ (776 cm^–1^). Based on the matrix elements in Table 4, it appears that such a shift is inherent to the intrinsic (one-mode) anharmonicity of the *Q*_3_ mode. The diagonal elements are FHF^–^ (1677 cm^–1^), ClHCl^–^ (979 cm^–1^), BrHBr^–^ (988 cm^–1^), and IHI^–^ (927 cm^–1^). These diagonal elements would correspond to the peak positions if each mode is treated anharmonically but is not coupled with the other modes. We note that these diagonal elements are higher than the corresponding predicted peak positions obtained from a fully coupled anharmonic treatment (Figure 3). This highlights the crucial role of the *n*_1_ν_1_ + ν_3_ combination bands in lowering the ν_3_ frequency. Hence, the present results affirm the vital role of the *Q*_1_ mode in modulating the *Q*_3_ mode’s frequency.^96^

### 3.4 Vibrational Adiabatic Model for the *Q*_1_ and *Q*_3_ Modes

The adiabatic model^12,74,98–106^ can be utilized to gain insights into studying the spectra of molecular systems that involve a high-frequency mode and a low-frequency mode. Such an approach has been used in the explanation and understanding of progression bands in hydrogen-bonded systems.^12,98–105^ In this work, we explore the insights gained by treating the slow symmetric X–H stretch (*Q*_1_) and fast asymmetric X–H stretch (*Q*_3_) modes in an adiabatic manner. To make the discussion simple, the adiabatic model is restricted to a two-dimensional (2D) case.

The 2D vibrational Hamiltonian has the form

9 $$Ĥ=-\frac{\hbar^{2}}{2\mu_{1}}\frac{\partial^{2}}{\partial Q_{1}^{2}}-\frac{\hbar^{2}}{2\mu_{3}}\frac{\partial^{2}}{\partial Q_{3}^{2}}+\mathrm{V̂}\left(Q_{1},Q_{3}\right)$$

We again note that the above Hamiltonian is a simplification of the Watson Hamiltonian.^78^ The vibrational angular momentum terms are neglected in the present treatment.

The key idea in the adiabatic model is that the *Q*_3_ transitions occur faster than the *Q*_1_ transitions. As a result, the slow *Q*_1_ motion and the fast *Q*_3_ motion can be treated separately. In the adiabatic model, the 2D vibrational Schrödinger equation is solved in two steps. In the first step, the Schrödinger equation for the fast mode (*Q*_3_) is solved at fixed values of *Q*_1_.

10 $$\left[-\frac{\hbar^{2}}{2\mu_{3}}\frac{\partial^{2}}{\partial Q_{3}^{2}}+\mathrm{V̂}\left(Q_{3};Q_{1}\right)\right]\phi_{n_{3}}\left(Q_{3};Q_{1}\right)=\varepsilon_{n_{3}}\left(Q_{1}\right)\phi_{n_{3}}\left(Q_{3};Q_{1}\right)$$

where V̂(*Q*_3_; *Q*_1_) denotes a potential energy curve along *Q*_3_ with *Q*_1_ as a parameter. Similarly, $\phi_{n_{3}}\left(Q_{3};Q_{1}\right)$ is the wave function for the *Q*_3_ mode with with *Q*_1_ as a parameter. The eigenvalues of the above Schrödinger equation depend on the slow mode’s coordinate (*Q*_1_), which can then be used as a potential energy for the slow mode’s motion.

11 $$\left[-\frac{\hbar^{2}}{2\mu_{1}}\frac{\partial^{2}}{\partial Q_{1}^{2}}+\varepsilon_{n_{3}}\left(Q_{1}\right)\right]\chi_{n_{3},n_{1}}\left(Q_{1}\right)=E_{n_{3},n_{1}}\chi_{n_{3},n_{1}}\left(Q_{1}\right)$$

where $E_{n_{3},n_{1}}$ is the adiabatic energy of the oscillator at state *n*_1_ and *n*_3_. The adiabatic wave function is then written as

12 $$\Psi_{n_{3},n_{1}}^{Adiabatic}\left(Q_{1},Q_{3}\right)=\chi_{n_{3},n_{1}}\left(Q_{1}\right)\phi_{n_{3}}\left(Q_{3};Q_{1}\right)$$

To predict a spectrum, a dipole moment function is needed. In order to keep the analysis simple, we will assume that the dipole moment is uncoupled and takes the form

13 $$\mathrm{\mu ̂}\left(Q_{1},Q_{3}\right)={\mathrm{\mu ̂}}_{1}\left(Q_{1}\right)+{\mathrm{\mu ̂}}_{3}\left(Q_{3}\right)$$

We believed that the coupling in the dipole can be ignored. Our previous work^74^ on Ar_n_H^+^ has shown that the potential coupling is way more important than the dipole coupling in the vibrational spectra.

Using the adiabatic wave function in eq 12, the transition dipole moment becomes

14 ⟨Ψn3′,n1′Adiabatic(Q1,Q3)|μ̂(Q1,Q3)|Ψn3′′,n1′′Adiabatic(Q1,Q3)⟩Q1,Q3=⟨ϕn3′(Q3)|μ3^(Q3)|ϕn3′′(Q3)⟩Q3⟨χn3′,n1′(Q1)|χn3′′,n1′′(Q1)⟩Q1

We note that the subscripts outside the bra-ket notation indicate the integration variables. The right-hand side in eq 14 contains two factors. The first factor corresponds to a transition dipole moment, with respect to *Q*_3_. Meanwhile, the second factor ${\left\langle \chi_{n_{3}^{\prime },n_{1}^{\prime }}\left(Q_{1}\right)\right|\chi_{n_{3}^{\prime \prime },n_{1}^{\prime \prime }}\left(Q_{1}\right)\rangle }_{Q_{1}}$ is the extent of overlap between two slow mode wave functions that belong to different fast mode states $n_{3}^{\prime }$ and $n_{3}^{\prime \prime }$ . As this picture is analogous to the well-known Franck–Condon principle in vibronic spectroscopy, we can therefore define the Franck–Condon factor for our purposes as follows:

15 $$S_{n_{3}^{\prime },n_{1}^{\prime }\leftarrow n_{3}^{\prime \prime },n_{1}^{\prime \prime }}={|{\langle \chi_{n_{3}^{\prime },n_{1}^{\prime }}\left(Q_{1}\right)|\chi_{n_{3}^{\prime \prime },n_{1}^{\prime \prime }}\left(Q_{1}\right)\rangle }_{Q_{1}}|}^{2}$$

We now examine the spectroscopic consequence of the adiabatic model. In the case of $n_{3}^{\prime \prime }=0$ and $n_{3}^{\prime }=1$ , the transition dipole moment for the fast mode will not vanish, since the direct product would correspond to a totally symmetric representation, $\Sigma_{g}^{+}$ . Since the integrated absorption intensity is proportional to the square of the transition dipole moment in eq 14, the progression band intensities are related to the Franck–Condon factors $S_{1,n_{1}^{\prime }\leftarrow 0,0}$ . In particular, the greater the overlap ${\left\langle \chi_{1,n_{1}^{\prime }}\left(Q_{1}\right)\right|\chi_{0,0}\left(Q_{1}\right)\rangle }_{Q_{1}}$ , the more probable and intense the transition.

We performed adiabatic treatment for each of the XHX^–^ using a 2D PES comprised of 169 grid points, which were low-dimensional cuts from the previously built 4D PES. As shown in Table 5, the Franck–Condon factor $S_{1,n_{1}^{\prime }\leftarrow 0,0}$ clearly modulates the intensity of the combination bands. For instance, the bright ν_3_ peak, which in the adiabatic model corresponds to the $\Psi_{1,0}^{Adiabatic}\leftarrow \Psi_{0,0}^{Adiabatic}$ transition, has the largest Franck–Condon factor across all XHX^–^, making it the most intense band among the progression. The *n*_1_ν_1_ + ν_3_ combination bands correspond to the $\Psi_{1,n_{1}}^{Adiabatic}\leftarrow \Psi_{0,0}^{Adiabatic}$ transitions. Their intensities are proportional to the Franck–Condon factor, $S_{1,n_{1}\leftarrow 0,0}$ .

**Table 5:** Comparison of Nonadiabatic and Adiabatic Treatment for the *Q*_1_ and *Q*_3_ Modes in XHX^–^

	Adiabatic Solution			Nonadiabatic Solution		Wave function overlap squared
Species	Peak (cm^–1^)	Franck–Condon Factor	Franck–Condon Intensity (km/mol)	Peak (cm^–1^)	Intensity (km/mol)	|⟨Ψ^Adiabatic^|Ψ^Nonadiabatic^⟩|^2^
FHF^–^						
ν_3_	1450	0.7328	2565	1454	2518	0.9997
ν_1_ + ν_3_	1983	0.2189	706	1986	738	0.9989
2ν_1_ + ν_3_	2508	0.0413	134	2509	141	0.9979
3ν_1_ + ν_3_	3042	0.0063	20	3038	22	0.9966
4ν_1_ + ν_3_	3623	0.0007	0	3612	3	0.9949
ClHCl^–^						
ν_3_	793	0.6059	4116	797	4043	0.9997
ν_1_ + ν_3_	1045	0.2957	1912	1047	1955	0.9989
2ν_1_ + ν_3_	1309	0.0819	536	1308	553	0.9981
3ν_1_ + ν_3_	1602	0.0146	100	1598	104	0.9973
4ν_1_ + ν_3_	1938	0.0017	11	1931	13	0.9961
BrHBr^–^						
ν_3_	840	0.5136	4083	841	4055	0.9999
ν_1_ + ν_3_	1012	0.3318	2621	1013	2635	0.9998
2ν_1_ + ν_3_	1886	0.1206	956	1186	964	0.9997
3ν_1_ + ν_3_	1375	0.0293	234	1375	237	0.9995
4ν_1_ + ν_3_	1588	0.0043	35	1589	35	0.9993
IHI^–^						
ν_3_	789	0.4083	4114	789	4104	0.99997
ν_1_ + ν_3_	911	0.3584	3621	911	3628	0.99992
2ν_1_ + ν_3_	1038	0.1724	1752	1038	1758	0.99985
3ν_1_ + ν_3_	1181	0.0513	525	1181	528	0.99979
4ν_1_ + ν_3_	1345	0.0087	90	1344	91	0.99972

To assess the performance of the adiabatic model, we have also performed a nonadiabatic treatment for each of the XHX^–^. Table 5 also shows the peaks and intensities from the nonadiabatic treatment. It is interesting to note that the peaks between the adiabatic and nonadiabatic solutions agree within 11 cm^–1^, which implies that the adiabatic eigenvalues are very close to those of their nonadiabatic counterparts. Furthermore, the trend in peak intensities predicted by the adiabatic and nonadiabatic treatments qualitatively agree with each other. To assess on how good the adiabatic wave functions are with respect to their nonadiabatic counterparts, the square of the overlap between their wave functions was calculated |⟨Ψ^Adiabatic^|Ψ^Nonadiabatic^⟩ |^2^. As shown in Table 5, the square of the overlaps is greater than 0.99, which means that the adiabatic solutions are almost identical to the nonadiabatic solutions.

Lastly, we note that the peak positions from a 2D treatment are higher than those from a 4D treatment (Figure 3). This is due to the fact that the 2D treatment had ignored the proton bending modes=creating an artificial confinement in the proton motion.

## 4 Summary and Conclusions

The XHX^–^ ions are the simplest ionic hydrogen-bonded species. These species contain only four vibrational degrees of freedom. As a result, a full-dimensional treatment can be performed. In the present work, a systematic study on the vibrational structure of XHX^–^ was made. The spectral features involve the bright ν_3_ band and the *n*_1_ν_1_ + ν_3_ combination bands. It was found that these combination bands are more pronounced as the halide gets heavier. For the first time, the quantum nature of the vibrational coupling between the ν_3_ and *n*_1_ν_1_ + ν_3_ bands was reported. The coupling elements were found to decrease from FHF^–^ to XHX^–^. The quantum nature of the *n*_1_ν_1_ + ν_3_ combination bands was examined using an adiabatic model, and it turns out that similar to several hydrogen-bonded systems, these combination bands can be viewed as Franck–Condon transitions between the ground and excited states of the asymmetric X–H stretch (*Q*_3_). Recently, Hirano and coworkers^107^ have examined the vibrational averaged structure for XeHXe^+^. They found that the average structure of XeHXe^+^ is asymmetric and bent. It might be worthwhile to reexamine the proton-bound noble gas dimers and hydrogen bihalides to see how these vibrationally averaged structures are sensitive with respect to the choice of coordinates. Although the present study had examined the anharmonic spectrum of these species in the gas phase, understanding the effects of the matrix is yet to be understood. As a result, it might be worthwhile to look at how the ion-matrix interaction would perturb the vibrational structure. Lastly, an attempt to understand the trend in the halide binding energies using ALMO-EDA was also discussed. It was found that the decrease in the halide binding energy to HX is a consequence of the increase in Pauli repulsion from FHF^–^ to IHI^–^. We hope that the present findings will encourage more gas-phase measurements for these species in the future.

## Supplementary Material



## Notes

Table S1: Energy decomposition analysis based on absolutely localized molecular orbitals (ALMO-EDA) for the bihalides at the ωB97M-V level of theory using the CCSD­(T)-optimized geometries; Table S2: Mulliken net atomic charges for the XHX^–^; Table S3: anharmonic frequencies (cm^–1^) for FHF^–^; Table S4: anharmonic frequencies (cm^–1^) for ClHCl^–^; Table S5: anharmonic frequencies (cm^–1^) for BrHBr^–^; Table S6: anharmonic frequencies (cm^–1^) for IHI^–^; Figure S1: qualitative MO for the sigma interaction in XHX^–^; Figure S2: anharmonic spectra for the BrHBr^–^ ion (PDF)
