# Supplementary material for: Theoretical Analysis of the n 1ν1 + ν3 Combination Bands in Hydrogen Bihalide Anions, XHX–, X = {F, Cl, Br, and I}
Source: J Phys Chem A. 2025 Nov 14;129(47):10848–58. doi: 10.1021/acs.jpca.5c05801 (PMC12670503; doi:10.1021/acs.jpca.5c05801)
Supplement: Supplementary file 1 [file jp5c05801_si_001.pdf]

Supporting Information for Theoretical Analysis of the $n_1\nu_1 + \nu_3$ Combination Bands in Hydrogen Bihalide Anions, XHX^-, X={F, Cl, Br, and I}

Jake A. Tan*,† and Jer-Lai Kuo*,‡

†*Department of Chemistry, University of West Florida, Pensacola, FL 32514, USA*

‡*Institute of Atomic and Molecular Science, Academia Sinica, Taipei 10617, Taiwan*

E-mail: jtan@uwf.edu; jlkuo@pub.iams.sinica.edu.tw

Table S1: Energy Decomposition Analysis based on Absolutely Localized Molecular Orbitals (ALMO-EDA) for the bihalides at the ωB97M-V level of theory using the CCSD(T) optimized geometries.The values are reported in kcal/mol.

Species	ΔE_{BIND}	ΔE_{GD}	$\Delta E_{ELEC}{}^{a}$	$\Delta E_{PAULI}{}^{a}$	ΔE_{DISP}	ΔE_{POL}	ΔE_{CT}
FHF^{-} [b]	-44.44	20.37	-72.27	80.92	-4.56	-45.02	-23.89
$ClHCl^{-}$ [b]	-23.43	18.39	-46.09	77.52	-4.92	-33.76	-34.58
$BrHBr^{-}$ [b]	-20.30	15.44	-42.79	78.66	-4.87	-33.28	-33.46
IHI^{-} [c]	-15.60	12.73	-38.32	85.47	-10.08	-27.88	-37.52

[a] $\Delta E_{DFFRZ} = \Delta E_{ELEC} + \Delta E_{PAULI}$

[b] Aug-cc-pVQZ basis set was used for H, F, Cl, and Br atoms.

[c] Aug-cc-pVQZ-PP basis set was used for I atoms.

Table S2: Mulliken Net Atomic Charges[a] for the XHX^{-}

Species	H	X
FHF^{-} [b]	0.575	-0.788
$ClHCl^{-}$ [b]	0.056	-0.528
$BrHBr^{-}$ [b]	-0.149	-0.425
IHI^{-} [c]	-0.250	-0.375

[a] Level of theory: ωB97M-V

[b] Aug-cc-pVQZ basis set was used for H, F, Cl, and Br atoms.

[c] Aug-cc-pVQZ-PP basis set was used for I atoms.

Table S3: Anharmonic frequencies (cm^{-1}) for FHF^- .

Assignment	Symmetry	ν (cm^{-1})	I (km/mol)
ν_1	Σ_g^+	585	0
ν_2	Π_u^+	1127	125
ν_3	Σ_u^+	1342	2521
$\nu_1 + \nu_3$	Σ_u^+	1862	696
$2\nu_1 + \nu_3$	Σ_u^+	2370	123
$3\nu_1 + \nu_3$	Σ_u^+	2881	17

Table S4: Anharmonic frequencies (cm^{-1}) for $ClHCl^-$.

Assignment	Symmetry	ν (cm^{-1})	I (km/mol)
ν_1	Σ_g^+	304	0
ν_2	Π_u^+	801	11
ν_3	Σ_u^+	974	1892
$\nu_1 + \nu_3$	Σ_u^+	1230	496
$2\nu_1 + \nu_3$	Σ_u^+	1504	85
$3\nu_1 + \nu_3$	Σ_u^+	1813	9

Table S5: Anharmonic frequencies (cm^{-1}) for $BrHBr^-$.

Assignment	Symmetry	ν (cm^{-1})	I (km/mol)
ν_1	Σ_g^+	191	0
ν_2	Π_u^+	716	2
ν_3	Σ_u^+	776	4092
$\nu_1+\nu_3$	Σ_u^+	943	2516
$2\nu_1+\nu_3$	Σ_u^+	1111	864
$3\nu_1+\nu_3$	Σ_u^+	1286	206
$4\nu_1+\nu_3$	Σ_u^+	1482	31

Table S6: Anharmonic frequencies (cm^{-1}) for IHI^- .

Assignment	Symmetry	ν (cm^{-1})	I (km/mol)
ν_1	Σ_g^+	135	0
ν_2	Π_u^+	631	0
ν_3	Σ_u^+	722	4329
$\nu_1+\nu_3$	Σ_u^+	841	3473
$2\nu_1+\nu_3$	Σ_u^+	962	1523
$3\nu_1+\nu_3$	Σ_u^+	1093	438
$4\nu_1+\nu_3$	Σ_u^+	1241	76

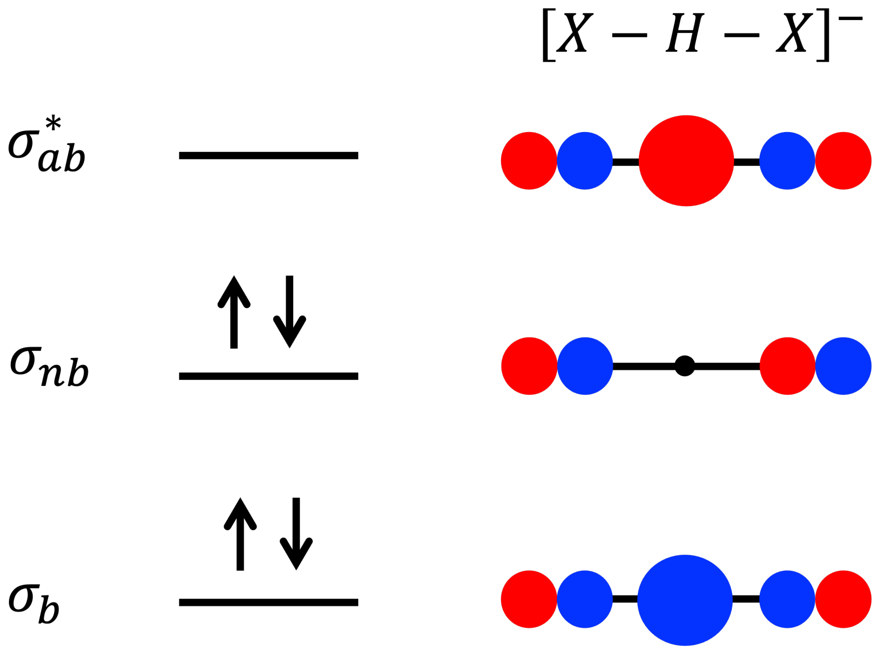

Figure S1: Qualitative MO for the sigma interaction in XHX-

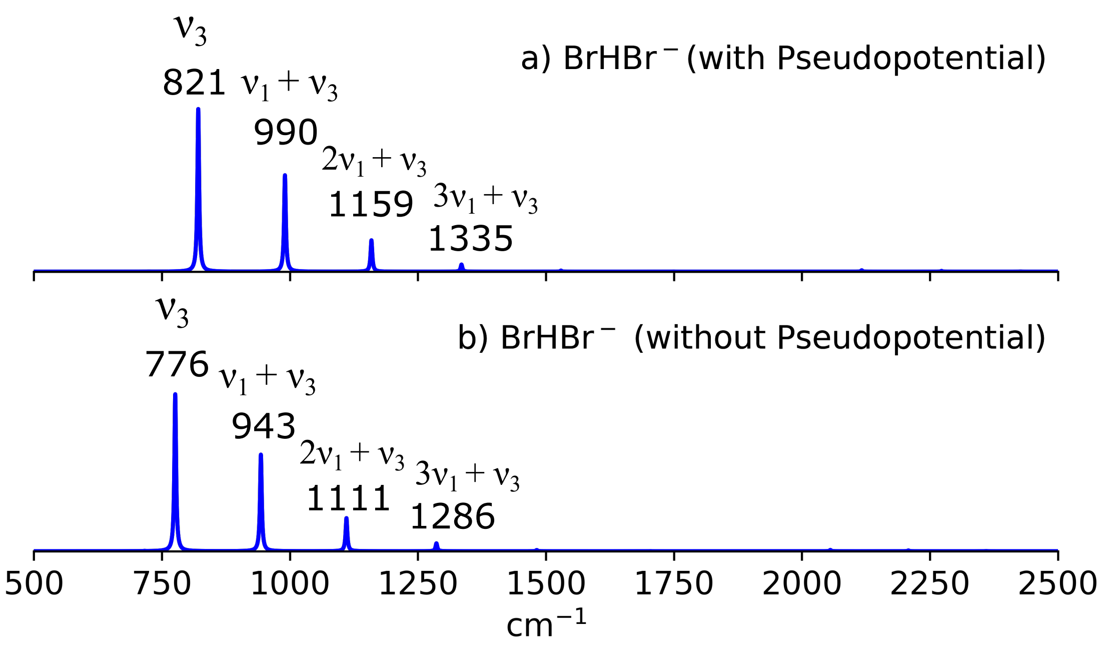

Figure S2: Anharmonic spectra for the BrHBr- ion a) with a pseudopotential from aug-cc-pVQZ-PP, and b) without a pseudopotential.
